# Reprogramming tumor cells with LCOR mRNA nanotherapy to enhance immunotherapy

**DOI:** 10.1002/1878-0261.70344

**Published:** 2026-10-15

**Authors:** Gabriel Serra‐Mir, Elena Haro‐Martínez, Sandra Blasco‐Benito, Chiara Cannatà, José Ángel Palomeque, Anna López‐Plana, Maria Sanz‐Flores, Marc Sabartés, Cristina Fornaguera, Toni Celià‐Terrassa

**Affiliations:** ^1^ Cancer Research Program Hospital del Mar Research Institute (HMRI) Barcelona Spain; ^2^ Institut Químic de Sarrià (IQS), Universitat Ramon Llull (URL) Barcelona Spain

**Keywords:** immunotherapy, mRNA therapy, phenotype constraint, transcription factor, triple‐negative breast cancer, tumor cell reprogramming

## Abstract

Transcription factors (TFs) are often considered undruggable due to their structural complexity. mRNA technologies can overcome this therapeutic limitation by enabling dynamic protein replacement therapies. Here, we explore the TF activity of LCOR (Ligand‐dependent corepressor), which suppresses tumor growth, at least in part by inducing the antigen presentation machinery (APM) in tumor cells and constraining cellular plasticity. These effects facilitate tumor immune recognition and immune‐mediated tumor eradication. We used poly β‐(amino esters) (pBAE) nanoparticles (NPs) for local delivery of *Lcor* mRNA in multiple breast cancer models. pBAE‐NPs efficiently encapsulate mRNA and facilitate cellular uptake, achieving optimal endosomal escape, resulting in high transfection efficiency *in vitro* and *in vivo*, restoring LCOR function in tumor cells and engaging their APM. In preclinical models, intratumoral delivery of *Lcor* mRNA reduced tumor growth. Importantly, the combination of *Lcor* mRNA‐loaded RH‐pBAE‐NPs with anti‐PDL1 or anti‐CTLA4 immunotherapies eradicated most of the tumors in our preclinical TNBC model. Overall, our nanotherapeutic strategy emerges as an innovative TF‐replacement therapy in a lock‐in/wipe‐out fashion, leveraging the immunogenic effects of LCOR to eradicate breast cancer tumors when combined with immunotherapy.

## Graphical abstract

LCOR (Ligand‐dependent corepressor) mRNA encapsulated in polymeric nanoparticles restores LCOR activity in tumor cells, constraining aggressive tumor cell states and increasing tumor immunogenicity. This reprogramming makes tumor cells more vulnerable to T‐cell attack and enhances their response to immune‐checkpoint immunotherapy. Graphical abstract created with BioRender.com.

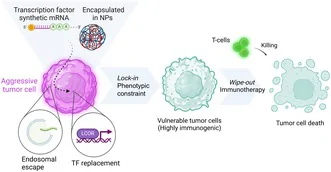

**Abbreviations**
^1^H‐NMR: proton nuclear magnetic resonance
3D: three‐dimensional
5‐moU: 5‐methoxyuracil
APM: antigen presentation machinery
ATCC: American Type Culture Collection
BSA: bovine serum albumin
CTLA4: cytotoxic T‐lymphocyte associated protein 4
Cy: cyanine
DBD: DNA‐binding domain
DLS: dynamic light scattering
DMEM: Dulbecco's modified Eagle's medium
DMSO: dimethyl sulfoxide
ECL: enhanced chemiluminescence
eGFP: enhanced green fluorescent protein
FBS: fetal bovine serum
Fluc: firefly luciferase
FRET: fluorescence resonance energy transfer
HTH: helix‐turn‐helix
i.p.: intraperitoneal
i.t.: intratumoral
ICB: immune‐checkpoint blockade
ICI: immune‐checkpoint inhibitor
IFN‐γ: interferon‐gamma
LCOR: ligand‐dependent corepressor
NP: nanoparticle
OVA: ovalbumin
pBAE: poly β‐(amino esters)
PBS: phosphate‐buffered saline
PDI: poly‐dispersity index
PDL1: programmed death‐ligand 1
SOC: standard‐of‐care
STR: short tandem repeat
TF: transcription factor
TNBC: triple‐negative breast cancer
TRD: treatment‐related death
UTR: untranslated regions

## Introduction

Transcription factors (TFs) have traditionally been considered undruggable, mostly because of their unstructured three‐dimensional (3D) shape. This feature makes it almost impossible to target TFs with small molecules, making it challenging to modulate their activity for clinical applications [1]. In recent years, mRNA has emerged as a promising therapeutic biomolecule, bypassing the traditional drawbacks of small molecules and achieving unprecedented potential for a range of clinical needs, as evidenced by COVID‐19 vaccines. mRNA technology is not only valuable for prophylactic vaccines but also for transient protein replacement therapies, which aim to substitute or restore specific proteins in target cells, opening new avenues in modulating TFs' activity to treat different disorders [2, 3, 4, 5].

Despite significant progress in mRNA technologies and nucleic acid delivery systems, achieving direct *in vivo* effective expression in target cells remains a major challenge in protein replacement therapies. Limitations mainly arise from mRNA stability in physiological fluids and low cell membrane penetration, which restrict success to the carrier and delivery system. Cationic polymer‐based nanovectors are among the most promising candidates for mRNA delivery [6]. Polyplex formation relies on electrostatic interactions between the cationic amine groups of polymers and the polyanionic mRNAs. This interaction leads to polynucleotide condensation into nanosized particles with a positive surface charge, which protects the nucleic acid and enhances stability. Poly β‐(amino esters) (pBAEs) polymers are a class of biocompatible and biodegradable polymers composed of amines and cleavable ester bonds in their backbones. These structures facilitate plasmatic cell penetration thanks to their cationic charges and endosomal escape due to protonation of tertiary amines at lower pH [7]. The use of oligopeptide‐end‐modified variants enhances their transfection capacity while preserving their nontoxic character [8, 9].

mRNA protein replacement approaches have been used in tumor cells to correct genetic alterations in preclinical models [2, 3, 8]. However, these approaches have not been used to modulate master TFs reprogramming cell phenotypes, such as differentiation, stemness, or immune evasion. We have previously described Ligand‐dependent corepressor (LCOR) as a breast cancer (BC) tumor cell differentiating factor reducing stemness, as well as a master TF regulating tumor cell immunogenicity in triple‐negative breast cancer (TNBC) and hormone receptor‐positive (HR+) breast cancer [10, 11, 12]. LCOR increases the immune detection and immune‐mediated killing by orchestrating the transcriptional activation of the antigen processing and presentation machinery (APM) independently of interferon‐gamma (IFN‐γ) signaling [11]. This observation is relevant since the downregulation of the APM is a major cause of resistance to immune‐checkpoint blockade (ICB) therapy [13, 14, 15], which is related to the loss of components of the IFN‐γ signaling pathway [16, 17, 18].

In breast cancer, ICB is part of the standard‐of‐care (SOC) for stage II/III TNBC and PD‐L1+ advanced TNBC [19, 20]. Only anti‐PD‐1/PD‐L1 therapy has been approved for TNBC, although others are under intense research, such as anti‐CTLA4 therapy, which has shown moderately positive results in recent clinical trials in early and advanced TNBC [21, 22]. However, in these patients, ICB leads to only a modest increase in response rates compared to chemotherapy alone [20]. Understanding and overcoming these barriers is critical for fully harnessing the potential of ICB therapies and improving outcomes for patients with TNBC. Here we demonstrate proof‐of‐concept for the therapeutic modulation of breast cancer cell phenotypes using pBAE‐nanoparticles (NPs) loaded with *Lcor* mRNA in preclinical mouse models. This approach showed exceptional efficacy in intratumoral settings, leading to tumor eradication when combined with PD‐L1 or CTLA4 blockade therapies. These innovative therapeutic strategies in a *lock‐in/wipe‐out* or *one‐two punch* fashion, open up new avenues for expanding the use of LCOR and TFs replacement therapies in immunotherapy and oncology.

## Material and methods

### Reagents

The reagents used for pBAE synthesis were bought from Sigma Aldrich® (St. Louis, MO, USA) and the oligopeptides CH3 (NH2‐Cys‐His‐His‐His‐COOH) and CR3 (NH2‐Cys‐Arg‐Arg‐Arg‐COOH), with a purity higher than 95%, from Shanghai Yaxian Chemical Co. Ltd (Jiading District, Shanghai, China). All synthetic mRNAs were supplied by Cellerna Bioscience (Baesweiler, North Rhine‐Westphalia, Germany), while the Cyanine3 and Cyanine5 NHS ester dyes (Cy3 and Cy5) were purchased from Lumiprobe GmbH (Westminster, MD, USA).

### Synthesis of end‐modified poly(β‐aminoester) polymers

The pBAE C6 polymer used was synthesized by a Michael addition reaction, as previously described [23]. Briefly, 5‐aminopentanol and 1‐hexylamine were added to 1,4‐butanediol diacrylate at the same ratio and using a slight excess of the latter (molar ratio of amine : diacrylate, 1 : 1.1). The reaction was carried out at 90 °C for 24 h. The polymer was further modified at its acrylate ends by the introduction of cationic oligopeptides, either CR_3_ or CH_3_ (molar ratio of polymer : peptide, 1 : 2.5). The mixture was stirred in dimethyl sulfoxide (DMSO) overnight at room temperature to later be precipitated in a mixture of diethyl ether/acetone. ^1^H‐NMR spectral characterization was performed to verify each synthesis step and the formation of the desired end structure.

### Nanoparticle formulation

The nanoparticles (NPs) were prepared using a mixture of polymer and nucleic acid, at a mass ratio of 25 : 1. Two starting solutions were prepared in sodium acetate buffer (12.5 mm, pH 5.2). One contained the mRNAs of choice at a final concentration of 0.5 mg·mL^−1^, while the other contained the polymer mixtures at a final concentration of 12.5 mg·mL^−1^. The polymer combinations used were C6‐CR_3_ and C6‐CH_3_ at a mass ratio of 60 : 40. The mRNA solution was pipetted into the polymer solution and thoroughly mixed for 15–20 s, followed by a 30‐min incubation at room temperature. Later, the solution was precipitated in the same volume of DEPC water as that of the NP formulation, and another equal volume of HEPES buffer (20 mm, pH 7.4) with 4% (w/v) of sucrose was added [24].

### mRNA labeling

The pH of the commercially available mRNA was first tested to determine whether it needed to be basified to achieve a neutral pH. The mRNA was mixed with Cy3 at a ratio of 10 : 3 (Cy3 : mRNA) (V_1_) and stirred for 2 h at room temperature in the dark. Later, 0.1 times the V_1_ of a sodium chloride solution (5 m) was added to the previous mixture (V_2_). Twice the V_2_ of cold pure ethanol was then added to the mixture and frozen overnight. The mixture was then centrifuged in a precooled centrifuge at 14 000× ***g*** at 4 °C for 15 min. The supernatant was then removed, and the pellet was rinsed with 70% ethanol and centrifuged again several times until the supernatant was clear. The pellet was then left to dry and characterized using the nanodrop setting of an Infinite M Plex TECAN Plate reader, where the concentration and purity of the mRNA and the amount of Cy3 in the sample were calculated.

### Polymer labeling

The C6‐CR_3_ at 100 mg·mL^−1^ was mixed with a solution of Cy5 at 10 mg·mL^−1^ in DMSO, taking 35 μL and 60 μL of each respectively. Triethylamine and DMSO were then added while protecting the reaction from light. The mixture was stirred for 20 h at room temperature. Next, it was precipitated in diethyl ether : acetone at a ratio of 7 : 3, vortexed, and centrifuged at 2254× ***g*** for 10 min. The supernatant was removed, and the pellet was rinsed twice with the same solution. The labeled polymer was left to dry for 24 h and then adjusted to 100 mg·mL^−1^ in DMSO. Later, the amount of Cy5 was tested through a calibration curve with several dilutions of Cy5 and read by fluorescence in an Infinite M Plex TECAN Plate reader.

C6‐CH3 labeling followed the same protocol but using 56 μL of C6‐CH3 and 100 μL of Cy5.

### mRNA complexation capacity and stability

The complexation capacity of the mRNA in the NPs was tested using the Ribogreen kit. The NPs were formulated at a ratio of 60 : 40 (C6‐CR_3_ : C6‐CH_3_) of the oligopeptide‐modified polymers with *e* *GFP* mRNA as a reporter gene. For the test, fresh NPs (free mRNA) were prepared and diluted in buffer and also in buffer with heparin, causing NP disruption and thus total exposure of the encapsulated mRNA (total mRNA). The different NP conditions were then compared to three standard curves (mRNA range, heparin, and NPs with heparin). Following the equation below, the percentage of encapsulated mRNA or encapsulation efficiency was calculated.

$$\mathrm{EE}\%=\left(1-\left(\frac{\text{free mRNA}}{\text{total mRNA}}\right)\right)\cdot 100$$

### Physicochemical characterization

NPs were characterized by Dynamic Light Scattering (DLS; ZetaSizer Nano ZS, Malvern). In this regard, their hydrodynamic diameter (nm) was measured, as well as their surface charge (mV) and poly‐dispersity index (PDI), by taking measures at room temperature with a 633 nm laser wavelength and a detector at an angle of 173°. The measurements were taken in triplicate, and the results are represented as mean ± standard deviation.

### Stability by fluorescence resonance energy transfer

NP stability was tested by fluorescence resonance energy transfer (FRET). To this end, the genetic material was labeled with Cy3 and the polymer with Cy5, thereby allowing the study of the complexation and structural organization of the NPs. Higher energy transfer indicates closer proximity between the fluorophores, thereby signifying the formation of a complexed NP. Conversely, a decrease in energy suggests the separation of the polymer and genetic material, leading to NP disassembly. FRET measurements were carried out using a TECAN Infinite M Plex microplate reader and a flat black 96‐well plate. This test was performed in phosphate‐buffered saline (PBS) at 25 °C, simulating laboratory stability, and in a solution of 50 mg·mL^−1^ of Bovine Serum Albumin (BSA) at 37 °C to simulate physiological stability.

### Cell culture

AT3 (RRID:CVCL_VN46), 4T07 (RRID:CVCL_B383), 66cl4 (RRID:CVCL_9721), 4T1 (RRID:CVCL_0125), and EpRAS cells were kindly provided by Dr. Yibin Kang (Princeton University). EO771 (RRID:CVCL_GR23), EMT6 (RRID:CVCL_1923), MDA‐MB‐231 (RRID:CVCL_0062), and MCF7 (RRID:CVCL_0031) were purchased from the American Type Culture Collection (ATCC; Manassas, VA, USA). The identity of the cell lines used in this study was validated by short tandem repeat (STR) profiling and matched to reference databases to confirm authenticity. Dulbecco's modified Eagle's medium (DMEM; ATCC) was used to culture cells. The cell culture medium was supplemented with 10% fetal bovine serum (FBS), Pen‐Strep (100 U·mL^−1^), and L‐Glutamine (100 μg·mL^−1^). Experiments were performed at 37 °C in 5% CO_2_ conditions and the normal level of O_2_ in a cell culture incubator. All cell lines were routinely tested for Mycoplasma contamination.

### Cellular uptake of dye‐labeled mRNA‐loaded NPs

To monitor the cellular NP uptake, Cy3‐*eGFP*‐mRNA‐Cy5‐NPs and Cy3‐*Δhth‐Lcor mutant*‐mRNA‐Cy5‐NPs were prepared. AT3 or 4T07 cells were first seeded in 96‐well plates at a density of 1 × 10^4^ cells per well and incubated at 37 °C in 5% CO_2_ for 24 h. They were then incubated with medium (DMEM) containing Cy3‐*eGFP*‐mRNA Cy5‐RH‐pBAE‐NPs or Cy3‐*Δhth*‐mRNA‐Cy5‐NPs for different times. The cells were then washed with PBS, resuspended using Trypsin‐0.25% EDTA, and processed for flow cytometry analysis or live cellular uptake monitoring. Differences in transfection efficacy are observed depending on the initial cell number and plate format used, underscoring the importance of maintaining a consistent μg mRNA‐NPs‐to‐cell number ratio across experiments.

### RT‐qPCR analysis

Total RNA was purified using the RNeasy Mini Kit (Qiagen; Venlo, Limburg, Netherlands) and reverse transcribed into cDNA using the High‐Capacity cDNA Reverse Transcription Kit (Life Technologies; Carlsbad, CA, USA). RT‐qPCR was performed using the LightCycler 480 SYBR Green I Master Mix (Roche; Basel, Basel‐Stadt, Switzerland). Data were collected using quantstudio 12 k flex software. mRNA expression was normalized by the expression of *Gapdh* (RT‐qPCR primers are listed in Table S1).

### Flow cytometry analysis

Flow cytometry data were collected on either a Fortessa Flow Cytometer (BD) using facsdiva v.9.0 software and analyzed by flowjo software v.10.8.1. Briefly, cells were trypsinized, washed twice with PBS, 5% FBS, and 0.5 mm EDTA, passed through a 70‐μm filter, and counted before proceeding with a general staining protocol using anti‐SIINFEKL (OVA) or anti‐H‐2K^d^ (Flow cytometry antibodies are listed in the Table S2).

### Immunoblotting

Protein extracts from cultured cells were lysed using RIPA buffer. Equal amounts of protein were determined using a bicinchoninic acid protein assay kit (Pierce/Thermo Scientific), following the manufacturer's instructions. After gel electrophoresis and protein transfer, membranes were blocked with 5% BSA in TBST for 1 h at room temperature with gentle shaking. Membranes were rinsed and then incubated overnight at 4 °C with appropriate primary antibodies (listed in the Table S2). The immunoreactive bands were visualized using an enhanced chemiluminescence (ECL) detection system (Merck; Rahway, NJ, USA). Data were collected using nine alliance q9 software in an UVITEC Cambridge system.

### Animals

Approximately 8‐week‐old female BALB/c and C57BL/6J mice were purchased from Charles River Laboratories and housed in a pathogen‐free animal facility at the Barcelona Biomedical Research Park. All animal procedures presented in this study were approved by the Ethical Committee for Animal Research of the Barcelona Biomedical Research Park and the *Departament de Medi Ambient i Habitatge de la Generalitat de Catalunya* (Catalan Government) under authorization number 10578. For all experimental procedures, euthanasia was applied once tumors reached 1500 mm^3^ or when animal health was compromised. Five animals (with the same genotype and sex) were maintained in individually ventilated Makrolon cages at a temperature of 22 ± 2 °C, with a relative humidity of 55 ± 5%, 12 h light : dark cycle starting at 08 : 00 am, and food and water *ad libitum*.

### Orthotopic tumor model preparation

For primary tumor models, 5 × 10^3^ 4T07 cells or 5 × 10^5^ AT3 cells were orthotopically injected into the MFP using 1 : 1 PBS : Matrigel (Corning). Tumor volume was measured twice weekly with digital calipers, and calculations were applied (π × length × width^2^/6). In ICB assays, once tumors reached 30–50 mm^3^, they were treated with either pBAE‐RH mRNA at a dose of 250 μg·kg^−1^ by intratumoral (i.t.) injection twice a week, anti‐PDL1 at a dose of 5 mg·kg^−1^ by intraperitoneal (i.p.) injection once a week, or their combination. On the other hand, mice were treated with anti‐CTLA4 with an initial dose of 5 mg·kg^−1^, followed by five doses at 0.5 mg·kg^−1^, by i.p. injection every 7 days, or their combination with pBAE‐RH mRNA at a dose of 250 μg·kg^−1^ by i.t. injection twice a week (all ICB antibodies are listed in the Table S2). In the therapeutic experiments, the *Δhth‐Lcor mutant* mRNA, which lacks the DNA‐binding domain, was used as the control mRNA. Animals were classified as responders (R) when tumor regression was observed, nonresponders (NR) when tumor progressed, or treatment‐related deaths (TRD) when premature death associated with repeated anti‐PD‐L1 administration prevented final tumor‐response assessment. Treatment‐related deaths were censored at the time of death in the tumor‐related survival analysis.

### Tissue immunofluorescence

Paraffin‐embedded tissue samples were cut into 3‐μm slices. After tissue dehydration and deparaffinization, antigens were retrieved with 0.5 m citrate, pH 6, in a pressure bath for 20 min. Tissue slices were incubated in blocking buffer (PBS, 2.5% Normal Goat Serum, 0.1% Triton X) and incubated overnight at 4 °C with primary antibody (listed in the Table S2). Tissue was washed, and fluorescent conjugated secondary antibodies were incubated for 1 h at room temperature. It was then washed again, and samples were mounted in DAPI medium (0100‐20; SouthernBiotech; Birmingham, AL, USA).

### Fluorescent *in situ* hybridization (FISH)

Paraffin‐embedded tissue samples were cut into 3‐μm slices. After tissue dehydration and deparaffinization, samples were treated for 10 min with hydrogen peroxide. Antigens were retrieved with 0.5 m citrate (pH 6) in a pressure bath for 20 min. Tissue slices were treated for 30 min with Protease solution at 40 °C. The RNAscope™ 2.5 HD detection kit was then used following the manufacturer's instructions (322360; ACD; Newark, CA, USA). *Lcor* mRNA was detected using mouse Lcor RNAscope™. Samples were washed and mounted in DAPI medium.

### pBAE‐NP transfection efficacy *in vivo*

Once tumors reached the desired volume, mice were euthanized and tumors were extracted. Samples were mechanically digested followed by a 30‐min enzymatic digestion with 2 mg·mL^−1^ Collagenase A and 0.5 mg·mL^−1^ DNAse I. Cells were passed through a 100‐μm strainer and incubated for 5 min at 37 °C in ACK lysing buffer. They were then passed through a 40‐μm strainer, counted, and processed for flow cytometry analysis. All gating strategies for Fig. 3 are summarized in Fig. S6.

### Isolation of tumor‐infiltrating immune cells and flow cytometric analysis

Harvested tumors were processed as the section above. Cells were incubated with FcγIII/II receptor CD16/CD32 (101302; BioLegend) and True‐Stain Monocyte blocking antibodies (426102; BioLegend; San Diego, CA, USA) for 10 min at 4 °C, followed by surface staining with antibodies for 20 min at 4 °C. For intracellular staining, fixable viability dye live/dead blue (L23105; Invitrogen, Thermo Fisher Scientific; Carlsbad, CA, USA) was used to stain dead cells. Cells were fixed and permeabilized with the Foxp3/Transcription Factor kit (005‐52 300; Invitrogen, Thermo Fisher Scientific) according to the manufacturer's protocol, followed by a 1‐h incubation at 4 °C with intracellular antibodies.

### Statistical analysis

The sample size of the animal studies was determined based on pilot experiments or previous studies. Experiments were performed with female mice of a similar age. Mice were randomized before cell injections and treatment allocation. Researchers were not blinded during experimentation and outcome since it was necessary to monitor each group. For all *in vivo* and *in vitro* experiments, independent biological replicates are indicated in the figure legends. Results are represented as mean ± S.E.M. Normality of data were checked using the Shapiro test and homoscedasticity using Bartlett's test. For comparisons between two groups, statistical significance was assessed using a *t*‐test or a Mann–Whitney *U*‐test. For multiple independent groups, one‐way ANOVA, the Kruskal–Wallis *H*‐test or multiple mixed‐effect analysis was used. For statistical significance. *P < 0.05, **P < 0.01, ***P < 0.001, ****P ≤ 0.0001. All experiments were reproduced in at least three independent biological replicates unless specified in the figure legend.

## Results

### mRNA delivery by pBAE‐NPs *in vitro*

pBAE‐NPs were formed by the electrostatic interaction of mRNA with the polymer, using a polymer combination of 60% arginine (R) and 40% histidine (H) oligopeptides as cationic end‐modified pBAEs (Fig. 1A), previously confirmed to be nontoxic [25]. NP synthesis yielded small nanometric and monodisperse particles (Fig. 1B and Fig. S1A) with high transfection efficiency *in vitro* and *in vivo* in several hosts [8, 26]. The efficiency of *eGFP* mRNA‐loaded RH‐pBAE‐NPs to transfect mRNA into different mouse breast cancer cells (AT3, 4T07, EO771, EMT6, 66cl4, EpRAS, and 4T1) was tested using NPs encapsulating *eGFP* mRNA, at a dose of 2.5 μg·mL^−1^. After 48‐h treatment, AT3 and 4T07 cells displayed the highest translated eGFP protein (Fig. S1B) after NPs uptake. In human models MDA‐MB‐231 and MCF7 cells, the NPs also showed high *eGFP* mRNA transfection efficiency (Fig. S1C). pBAE‐NPs efficiently encapsulated 60% of *Lcor* mRNA (Fig. S1D). To determine the endosomal escape of these NPs across the different cell lines, we fluorescently labeled the polymer and mRNA with Cyanine (Cy)5 and Cy3, respectively, allowing to monitor endosomal escape and mRNA translation kinetics by flow cytometry and fluorescence microscopy. Time course analysis by flow cytometry and fluorescence microscopy analysis of Cy5, Cy3, and eGFP fluorescence showed higher uptake and translation of *eGFP* mRNA in 4T07 cells (Fig. 1C,D), with the expected dose‐dependent effect (Fig. S1E), and flow cytometry of eGFP positivity percentage (Fig. 1E). Note that these percentages are influenced by differences in cell proliferation rates over the course of the culture period. These results demonstrate that tumor cells can uptake the mRNA‐loaded RH‐pBAE‐NPs and that these particles can escape the endosomal system and deliver the mRNA to the cytoplasm for proper translation of the protein.

**Fig. 1:**
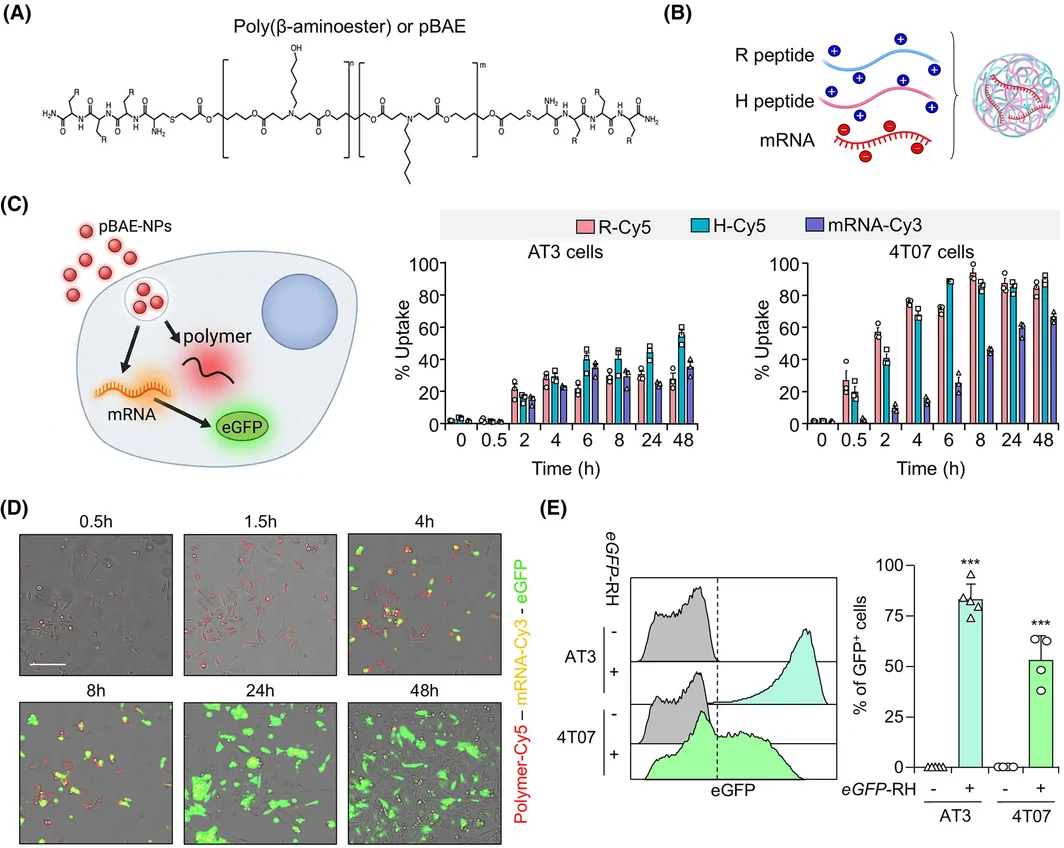
pBAE‐NPs efficiently transfect breast cancer cell lines with mRNA *in vitro*. (A) Poly β‐(amino ester) (pBAE) polymers are composed of cleavable ester bonds and amines. The R‐terminal can be an arginine (R) or histidine (H) oligopeptide. (B) Polyplex assembly is based on interactions between the positively charged amine groups of polymers – (arginine (R) or histidine (H)) – and the negatively charged mRNA. This interaction leads to condensation into nanosized particles, which protect the nucleic acid. (C) Flow cytometry analysis of the percentage of pBAE‐NP uptake over time in TNBC 4T07 and AT3 mouse cell lines. mRNA was labeled with cyanine (Cy)‐3, whereas the polymers were labeled with Cy5. Data represent the mean % of total gated cells + S.E.M. of *N* = 3 independent experiments. (D) Fluorescence microscopy images of TNBC 4T07 cells treated with RH‐pBAE‐NPs labeled with Cy3 (*eGFP*‐mRNA) and Cy5 (R‐modified polymer) over time. Scale bar = 50 μm. Images are representative of *N* = 2 independent experiments. (E) Flow cytometry analysis of the percentage of TNBC 4T07 and AT3 cells positive for eGFP expression from 5 μg of *eGFP* mRNA‐loaded RH‐pBAE‐NPs. Data represent the mean % of total gated cells + S.E.M. of *N* = 4 and 5 independent experiments for 4T07 and AT3, respectively. Statistical significance: ***P < 0.001, by one‐way ANOVA test in C and Kruskal–Wallis *H*‐test followed by Dunn's *post hoc* test in E.

### *Lcor* mRNA delivery by pBAE‐NPs and TF functionality in tumor cells *in vitro*

To test the dynamics and ability of our system to deliver TFs to tumor cells, we treated AT3 and 4T07 cells *in vitro* with pBAE‐NPs loaded with *Lcor* mRNA. Synthetic *Lcor* mRNA contained a Cap1, 5′ and 3′ untranslated regions (UTR) and a standard polyA tail (Fig. S2A), and all uracils were replaced for 5‐methoxyuracil (5‐moU) to avoid immunogenic reactions [27, 28]. The *Δhth*‐*Lcor* mutant mRNA was used as negative control (Fig. S2A). This mutant lacks the HTH DNA‐binding domain (DBD), which is responsible for DNA binding to regulatory regions to induce gene expression [10, 11]. First, we measured and detected high levels of *Lcor* mRNA by qRT‐PCR (Fig. 2A), increasing in a dose‐dependent manner (Fig. S2B), as expected. RH‐pBAE NPs were stable at 25 °C for 24 h (Fig. S2C). In contrast, under conditions simulating the physiological environment (37 °C), a decrease in FRET signaling was detected as the incubation time increased, indicating disassembly of the NPs after 2 h (Fig. S2C). The rapid disassembly of NPs within 2 h at 37 °C is a crucial feature of our delivery system, as it facilitates timely release of the mRNA payload inside the target cells, which is essential for efficient translation and biological activity. After treatment of the cells with the NPs *in vitro*, we detected cellular uptake of mRNA, with a peak at 6 h, which was maintained up to 36 h in culture (Fig. 2A), thereby indicating a rapid uptake and release of mRNA and a transient cell expression. Importantly, to measure the translated product of the *Lcor* mRNA delivered, we performed western blot analysis. High levels of LCOR protein were observed in tumor cells after 12 h of *Lcor* mRNA‐loaded RH‐pBAE‐NPs treatment (Fig. 2B). These findings indicate that this technology can deliver synthetic *Lcor* mRNA into tumor cells, where it is efficiently translated into LCOR protein.

**Fig. 2:**
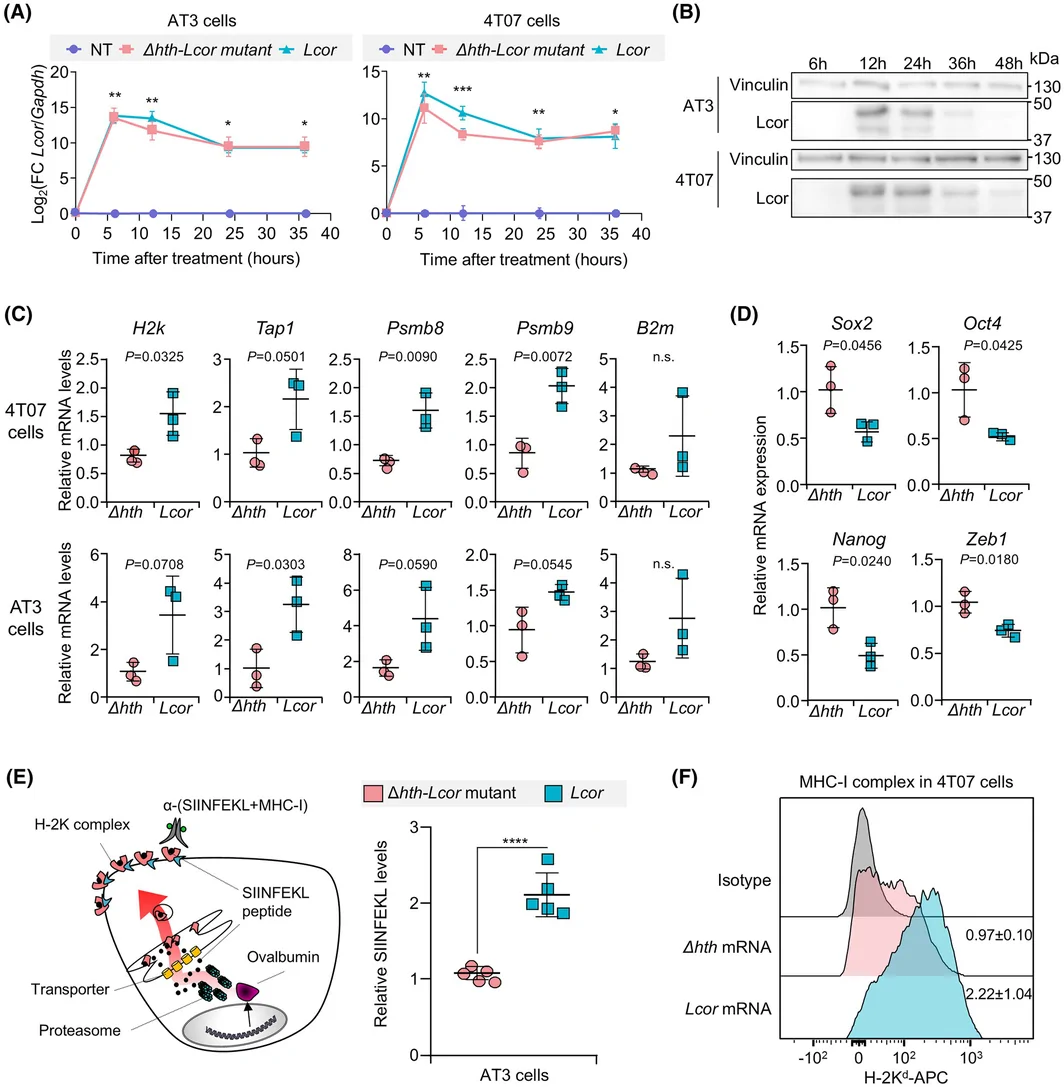
*Lcor* mRNA delivery by pBAE‐NPs and TF functionality in tumor cells *in vitro*. (A) RT‐qPCR analysis of *Lcor* mRNA levels over time in TNBC AT3 and 4T07 cells treated with 5 μg of the indicated mRNA‐loaded RH‐pBAE‐NPs. Data are represented as Log_2_(Fc) to the normalized expression of nontreated control condition. Expression levels were normalized to *Gapdh*. Data represent the mean ± S.E.M. of *N* = 4 and 3 independent experiments for AT3 and 4T07 cell lines, respectively. (B) Western blot image of TNBC AT3 and 4T07 cells treated with *Lcor* mRNA‐loaded RH‐pBAE‐NPs for 6, 12, 24, 36, and 48 h. Detection of Lcor and Vinculin (loading control). Western blots are representative of *N* = 3 independent experiments. (C) RT‐qPCR analysis of APM genes in TNBC 4T07 (top) and AT3 (bottom) cells treated with 5 μg of the indicated mRNA‐loaded RH‐pBAE‐NPs for 36 h. Data are represented as Fc to the normalized expression of nontreated control condition. Expression levels were normalized to *Gapdh*. Data represent the mean ± S.E.M. of *N* = 3 independent experiments. (D) RT‐qPCR analysis of stemness genes in 4T07 cells treated with 5 μg of the indicated mRNA‐loaded RH‐pBAE‐NPs for 24 h. Data are represented as Fc to the normalized expression of nontreated control condition. Expression levels were normalized to *Gapdh*. Data represent the mean ± S.E.M. of *N* = 3 independent experiments. (E) Flow cytometry analysis of antigen presentation levels in AT3‐OVA cells treated with 5 μg of the indicated mRNA‐loaded RH‐pBAE‐NPs for 36 h. AT3‐OVA cells were stained for SIINFEKL‐H‐2K^b^ complexes to assess peptide–Mhc I presentation. Expression levels were normalized to nontreated control condition. Data represents the mean ± S.E.M. of *N* = 5 independent experiments. (F) Representative flow cytometry fluorescence spectra showing antigen presentation levels in 4T07 cells treated with the indicated mRNAs. 4T07 cells were stained for H‐2Kᵈ to assess peptide–Mhc‐I presentation. Histograms show mean fluorescence intensity (MFI) distributions for the indicated treatment conditions. Average fold change of the MFI ± SD is shown for each condition. (A, C, D, E) Statistical significance; *P < 0.05, **P < 0.01, ***P < 0.001, ****P ≤ 0.0001 by linear mixed model analysis with Geisser–Greenhouse correction, followed by Tukey's multiple comparisons test for A, unpaired two‐tailed Student's *t*‐test in C and D, and Kruskal–Wallis *H*‐test followed by Dunn's *post hoc* test in E.

Next, to assess whether LCOR can restore its function, we challenged Lcor function as suppressor of stemness and as APM inducer [10, 11, 12]. The results showed a functional effect of the NP treatment mediated by LCOR‐inducing APM genes in AT3 and 4T07 cell lines after 36 h of treatment (Fig. 2C). In addition, we tested the previously described ability of Lcor to repress stemness by RT‐qPCR analysis. Remarkably, 24 h after a single dose of *Lcor* mRNA significantly reduced the expression of stem cell gene programs such as *Sox2*, *Oct4*, *Nanog*, and *Zeb1* (Fig. 2D). In addition, we used a reporter with the Nanog promoter which has binding sites for Sox2, Oct4 and Nanog to measure their transcriptomic activity reported by Ds‐eGFP expression [29]. Treatment with *Lcor* mRNA reduced both the percentage of Ds‐eGFP+ cells and the mean fluorescence intensity in 4T07 cells (Fig. S2D), showcasing the ability of Lcor mRNA therapy (with a single dose) to suppress stemness‐associated cell states. Furthermore, *Lcor* mRNA showed a trend toward increasing other IFN targets such as, *Pd‐l1* (*Cd274*) expression, while no changes were observed in *Cd80* nor *Cd86* (Fig. S2E). Together these results further support the reprogramming capacity of *Lcor* mRNA therapy, reconfiguring tumor cell states toward differentiation and high immunogenic cells.

Next, we used AT3 cells that constitutively overexpress ovalbumin (OVA). In these cells, OVA is cleaved, generating the SIINFEKL antigen peptide presented in the H‐2K^b^ context. This can be used to measure overall APM activity using the anti‐SIINFEKL antibody via flow cytometry [30]. The results showed an increase of OVA‐SIINFEKL presentation, indicating the enhanced activity of the APM induced by the *Lcor* mRNA‐loaded RH‐pBAE‐NPs (Fig. 2E). When comparing the effect of *Lcor* mRNA on 4T07 cell line, the results did not reach significance (Fig. 2F). Consistently, measuring Mhc‐I molecules on the cell surface of AT3 and 4T07 cells showed an increase of Mhc‐I levels. Overall, these results demonstrate the efficiency of our mRNA therapy nanotechnology to rescue the function of the LCOR TF in inducing tumor cell immunogenicity and thus modulating tumor phenotypes [11].

### pBAE‐NPs efficiently deliver mRNA into breast cancer tumors *in vivo*

To test the capacity of pBAE‐NPs to deliver mRNA to tumor tissue from preclinical models, we encapsulated *eGFP* or *Firefly Luciferase* (*FLuc*) mRNA for fluorescence or bioluminescence (BLI) detection of recipient cells. We generated primary tumors by orthotopic mammary fat pad (MFP) injection of 4T07 cells into immunocompetent BALB/c mice. When tumors reached 40–50 mm^3^, we treated them intratumorally with pBAE‐NPs loaded with 5 μg of synthetic *FLuc* or *eGFP* mRNA. We detected BLI at 3 h, meaning that tumor cells had taken up the mRNA‐loaded NPs and translated a luciferase active protein within 3 h. Luciferase expression peaked 10 h after administration (Fig. S3A). In addition, we injected *eGFP* mRNA‐loaded RH‐pBAE‐NPs into mice bearing 4T07‐mCherry^+^ tumors. After 6 h, we harvested and digested the tumor tissues to evaluate, by eGFP fluorescence using flow cytometry, showing how tumor cells, and also stromal cells to a lesser extent, incorporated and translated the mRNA (Fig. 3A), thereby indicating substantial transfection capacity *in vivo* after one dose of 5 μg of mRNA‐loaded NPs.

**Fig. 3:**
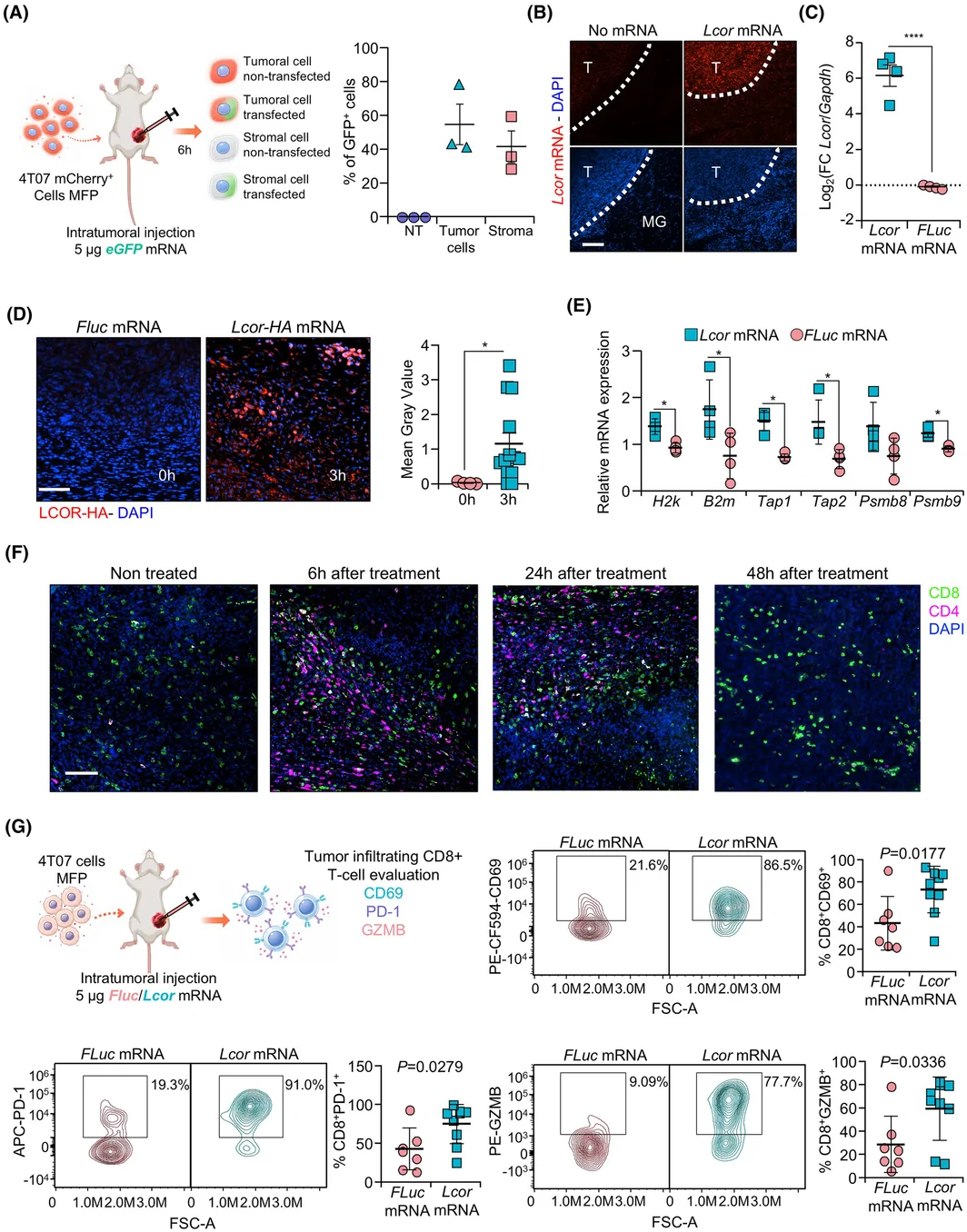
pBAE‐NPs efficiently deliver mRNA into breast tumors *in vivo*. (A) Flow cytometry analysis of *eGFP* mRNA‐loaded RH‐pBAE‐NPs uptake in tumor (mCherry^+^) and stromal (mCherry^−^) cells (gated from Live cells, Fig. S6A). Once tumors reached 40–50 mm^3^, tumors were intratumorally treated with 5 μg of *eGFP* mRNA‐loaded RH‐pBAE‐NPs. After 6 h, the tumors were harvested, mechanically and enzymatically digested for single‐cell suspension, and analyzed. Data represent the % of eGFP^+^ cells in each population. Results are shown as mean ± S.E.M. from *N* = 3 mice per group. (B) Representative fluorescence images of *Lcor* mRNA detected by *in situ* hybridization (ISH) (red) and nuclei stained with DAPI (blue). After reaching a size of 40–50 mm^3^, tumors formed by 4T07 cells implanted in the mammary fat pad of BALB/c mice were intratumorally treated with 5 μg of *Lcor* mRNA‐loaded RH‐pBAE‐NPs. High‐density nuclear regions correspond to tumor areas (T), whereas low‐density nuclear regions correspond to adjacent mammary gland (MG) tissue. Scale bar = 40 μm. Images are representative of *N* = 4 mice per condition. (C) RT‐qPCR analysis of *Lcor* mRNA levels in tumors formed by 4T07 cells implanted in the MFP of BALB/c mice. After reaching a size of 40–50 mm^3^, tumors were intratumorally treated with 5 μg of *Lcor* mRNA‐loaded RH‐pBAE‐NPs and harvested 24 h post‐treatment. Data are represented as Log_2_(Fc) to the normalized expression of *FLuc* mRNA treated control condition. Expression levels were normalized to *Gapdh*. Data represent the mean ± S.E.M. of *N* = 4 mice per group. (D) Representative fluorescence images of Lcor‐HA protein detected with anti‐HA antibody 3‐h post‐treatment. Nuclei are stained with DAPI (blue). After reaching a size of 40–50 mm^3^, tumors formed by 4T07 cells implanted in the mammary fat pad of BALB/c mice were intratumorally treated with 5 μg of *Lcor‐HA* mRNA‐loaded RH‐pBAE‐NPs. Quantification of fluorescence intensity was determined by imagej, showing the mean gray value. Data are shown as mean ± S.E.M. of 1 and 4 images per mouse for control and *Lcor‐HA* mRNA, respectively. Scale bar = 30 μm. (E) RT‐qPCR analysis of APM genes in tumors formed by 4T07 cells implanted in the mammary fat pad of BALB/c mice. After reaching a size of 40–50 mm^3^, tumors were intratumorally treated with 5 μg of indicated mRNA‐loaded RH‐pBAE‐NPs and then harvested 24‐h post‐treatment. Data represents the Fc of APM genes, normalized to *Gapdh*, shown as mean ± S.E.M. from *N* = 4 mice per group. (F) Representative immunofluorescence images of 4T07 tumor sections collected from untreated mice or at 6, 24, and 48 h after intratumoral administration of 5 μg of *Lcor* mRNA‐loaded RH‐pBAE‐NPs. Sections were stained for CD4 (magenta), CD8 (green), and DAPI (blue). Scale bar = 30 μm. Images are representative of *N* = 3, 4, 4, and 3 mice for the nontreated, 6, 24, and 48 h, respectively. (G) Flow cytometric analysis of activation (CD69), cytotoxic (GZMB), or exhausted (PD‐1) infiltrating CD8^+^ T cells from 4T07 tumors 24 h after treatment with 5 μg of *FLuc* or *Lcor* mRNA‐loaded RH‐pBAE‐NPs (gated from CD45 + CD8+, Fig. S6B). Results are shown as mean ± S.E.M from *N* = 7 animals per group. (C–E, G): Statistical significance: *P < 0.05, ****P < 0.0001, by Mann–Whitney *U*‐test in C–E, and unpaired two‐tailed Student's *t*‐test in G.

Next, we tested the detection of *Lcor* mRNA delivery *in vivo*. To this end, we used specific *Lcor* probes for tissue *in situ* hybridization (ISH) for *Lcor* mRNA detection and immunofluorescence (IF) for LCOR protein detection. After local intratumoral administration of 5 μg of *Lcor* mRNA‐loaded RH‐pBAE‐NPs when tumors reached 40–50 mm^3^ (Fig. S3B), we observed a rapid increase in *Lcor* mRNA in the tumor tissue (Fig. 3B). Consistently, similar results were obtained by RT‐qPCR (Fig. 3C). To unravel the protein dynamics, we used synthetic *Lcor* mRNA including the HA tag sequence to generate LCOR‐HA protein and uniquely detect the ectopic protein using anti‐HA by IF (Fig. 3D). Importantly, we validated that *Lcor* mRNA therapy led to increased APM genes in 4T07 cells (Fig. 3E) and AT3 cells (Fig. S3C,D).

To further functionally validate the tumor reconfiguration and immune microenvironment impact induced by *Lcor* mRNA‐loaded RH‐pBAE‐NPs treatment *in vivo*, 4T07 tumors were collected at different time points after administration. After *Lcor* mRNA‐loaded RH‐pBAE‐NPs administration, increasing *Lcor* mRNA levels (Fig. S3E) and increasing T‐cell infiltration were detected up to 24‐h post‐treatment, while decreasing after 48 h after treatment (Fig. 3F). To further characterize the T‐cell activity induced by *Lcor* mRNA‐loaded RH‐pBAE‐NPs, we performed flow cytometry‐based immune profiling of 4T07 tumors after treatment. Although no significant differences were observed in the overall abundance of CD4^+^ or CD8^+^ T cells at 24 h (Fig. S3F), tumors treated with *Lcor* mRNA‐loaded RH‐pBAE‐NPs showed a significant increase in the proportion of activated CD8+ T cells expressing CD69, GZMB, and PD1 compared with control‐treated tumors (Fig. 3G). These findings suggest that, beyond transiently promoting lymphocyte infiltration, *Lcor*‐mRNA‐therapy enhances T‐cell activation and acquisition of an effector‐like phenotype within the tumor microenvironment.

### *Lcor* mRNA nanotherapy synergizes with immune‐checkpoint inhibitors in preclinical models

To test the efficacy of the TF‐replacement therapy using *Lcor* mRNA‐loaded RH‐pBAE‐NPs, we first generated 4T07 breast primary tumors in immunocompetent BALB/c mice. Once tumors reached 40–50 mm^3^, *Lcor* mRNA‐loaded RH‐pBAE‐NPs were administered at a dose of 250 μg·kg^−1^ by intratumoral (i.t.) injection twice a week. In addition, we combined the *Lcor* mRNA therapy with the anti‐PDL1 immune‐checkpoint inhibitor (ICI), at a dose of 5 mg·kg^−1^, by intraperitoneal (i.p.) injection once a week (Fig. 4A). Results point toward *Lcor* mRNA‐loaded RH‐pBAE‐NPs monotherapy being sufficient to reduce 4T07 tumor growth (Fig. 4B, and Fig. S4B); however, the differences did not reach statistical significance. Importantly, the combination of *Lcor* mRNA‐loaded RH‐pBAE‐NPs with anti‐PDL1 therapy not only reduced tumor growth but also led to tumor eradication in 5 out of 7 mice (Fig. 4C). Regarding toxicity, 3 mice experienced premature death due to anti‐PDL1 toxicity, 2 mice in *Δhth* mRNA + anti‐PDL1 and one in *Lcor* mRNA + anti‐PDL1 (Fig. 4D), as previously reported in Balb/c mice bearing tumors from 4T1 series [31], however the combined *Lcor* mRNA‐loaded RH‐pBAE‐NPs plus anti‐PDL1 therapy did not have additional adverse immune effects, and mouse weights were similar across conditions during the treatment (Fig. S4A). The combination treatment also significantly increased tumor disease‐related survival, with treatment‐related deaths censored at the time of death (Fig. 4E). These results indicate that our *Lcor* mRNA nanotherapy synergized with anti‐PDL1 and overcame resistance in 4T07 tumors.

**Fig. 4:**
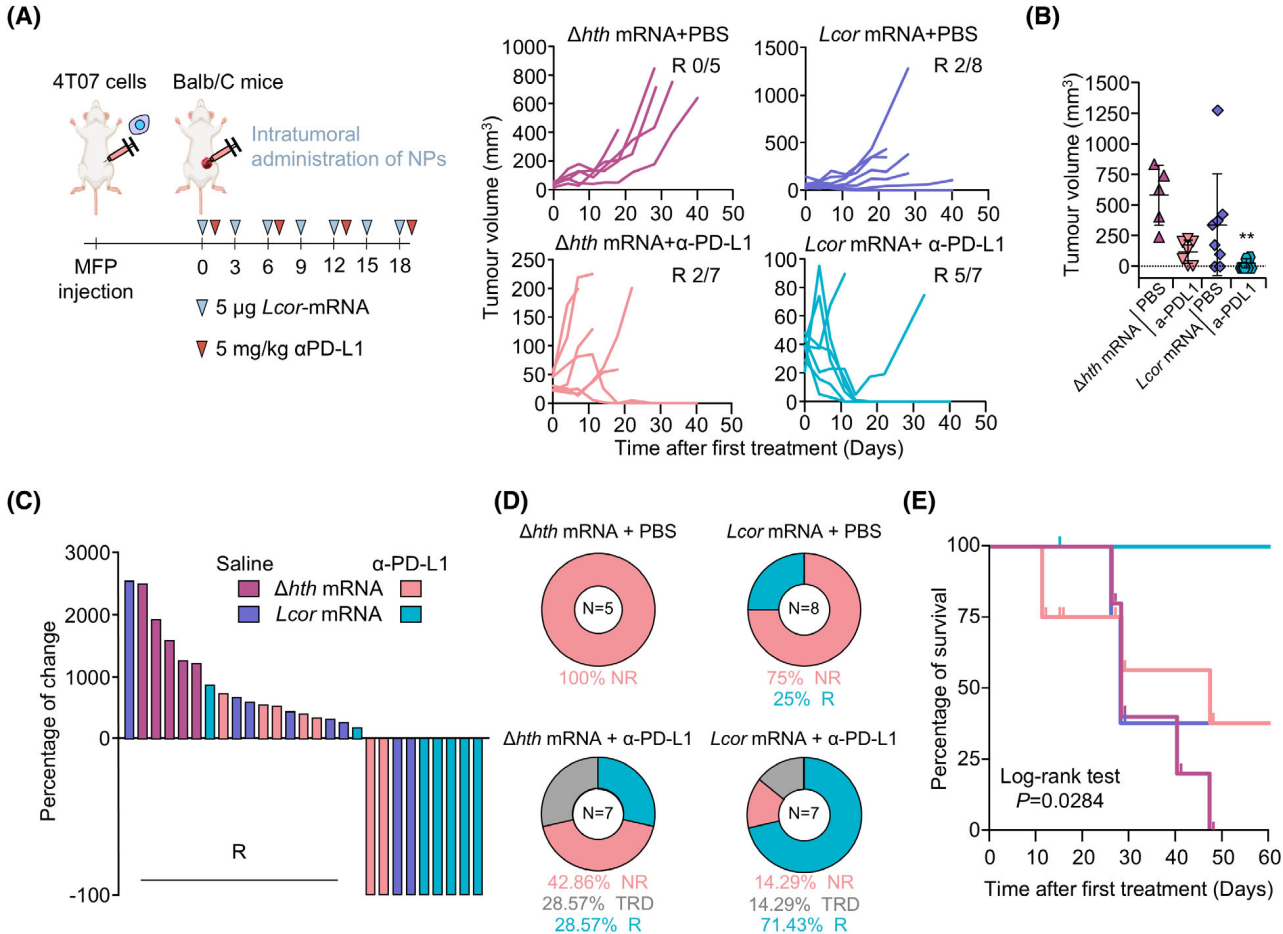
*Lcor* mRNA nanotherapy synergizes with anti‐PD‐L1 in preclinical models. (A) Tumor volume over time following the specified treatments. Mice were implanted with 4T07 cells in the mammary fat pad and treated with 5 μg of the indicated mRNA‐loaded RH‐pBAE‐NPs intratumorally twice a week, and 5 mg·kg^−1^ of anti‐PD‐L1 intraperitoneally once a week. (B) Tumor volume at the endpoint of the experiment for each treatment group. Data represent mean ± S.E.M. from *N* = 5‐8 mice per group. Statistical significance: **P < 0.01, by Kruskal–Wallis *H*‐test, followed by Dunn's *post hoc* test; *P* = 0.0028. (C) Waterfall plot of the percentage change in tumor volume for each mouse. (D) Donut plots showing the outcome of all animals assigned to each treatment group. Animals were classified as responders (R), nonresponders (NR), or treatment‐related deaths (TRD). R indicates tumor regression, NR indicates tumor progression, and TRD indicates premature death associated with repeated anti‐PD‐L1 administration before final tumor‐response assessment. Cyan indicates R, pink indicates NR, and gray indicates TRD. (E) Kaplan–Meier survival curves with statistical analysis by log‐rank test. *P* = 0.0284.

We extended our therapeutic approach to combine our *Lcor* mRNA‐loaded RH‐pBAE‐NPs therapy with the ICI anti‐CTLA4. Mice were subjected to MFP implantation of 4T07 cells. When primary tumors reached 40–50 mm^3^, the animals received either *Lcor* mRNA‐loaded RH‐pBAE‐NPs at a dose of 250 μg·kg^−1^ by i.t. injection twice a week alone or in combination with anti‐CTLA4, at an initial dose of 5 mg·kg^−1^, followed by five doses at 0.5 mg·kg^−1^ by i.p. injection every 7 days (Fig. 5A). Remarkably, all the mice treated with the combination therapy showed a complete response (Fig. 5A–C and Fig. S5A), and thus 100% survival (Fig. 5D).

**Fig. 5:**
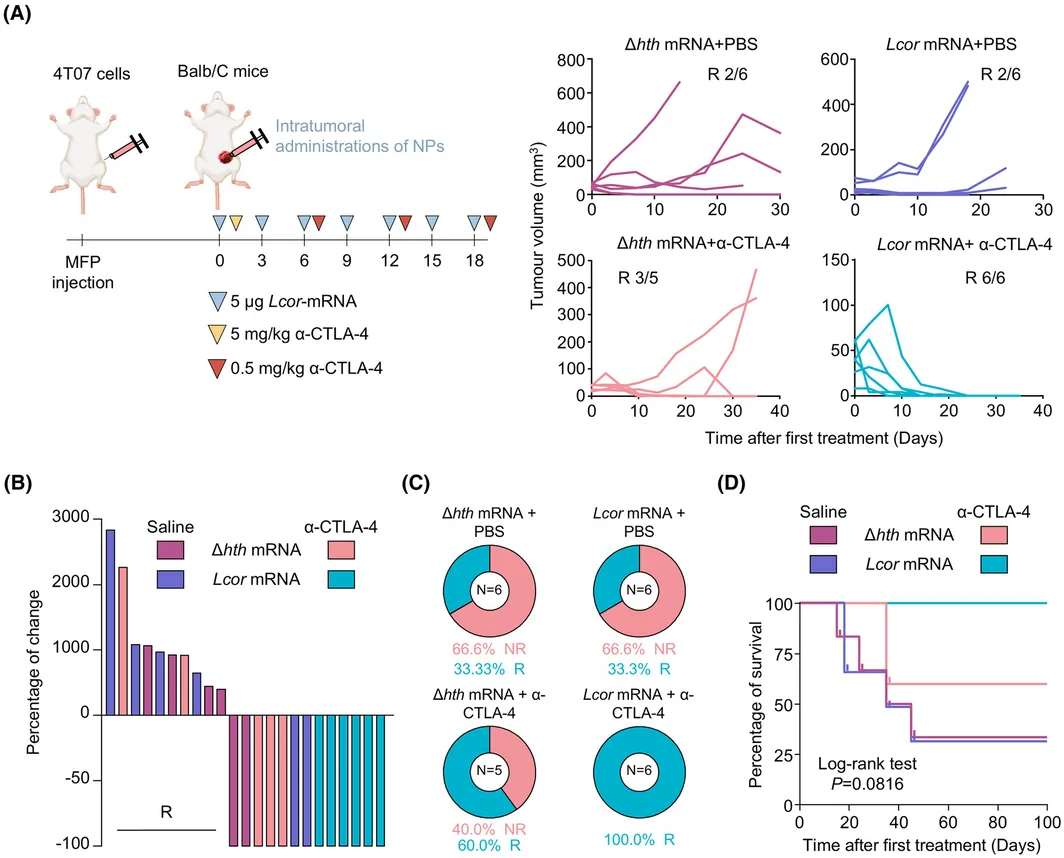
*Lcor* mRNA nanotherapy synergizes with anti‐CTLA4 in preclinical models. (A) Tumor volume over time following the specified treatments. Mice were implanted with 4T07 cells in the mammary fat pad and treated with 5 μg of the indicated mRNA‐loaded RH‐pBAE‐NPs intratumorally twice a week, and 5 mg·kg^−1^ of anti‐CTLA4 intraperitoneally for the first dose, followed by 0.5 mg·kg^−1^ for subsequent doses. (B) Waterfall plot of the percentage change in tumor volume for each mouse. Data are shown from *N* = 5–6 mice per group. (C) Donut plots showing the proportion of responder and nonresponder mice in each treatment group. Pink indicates nonresponder/progressive tumors and cyan indicates responder/regressing tumors. (D) Kaplan–Meier survival curves with statistical analysis by log‐rank test. *P* = 0.0816.

Overall, these results demonstrate the potential of our strategy in leveraging mRNA therapy to rescue the LCOR TF and reconfigure tumor phenotypic properties. The combination of *Lcor* mRNA‐loaded RH‐pBAE‐NPs with different ICIs showed high efficiency in preclinical models, thus supporting the potential of local delivery of *Lcor* mRNA‐loaded RH‐pBAE‐NPs in combination with ICIs as a promising immunotherapeutic strategy for potential future clinical applications.

## Discussion

Here we provide evidence of the capacity of nanomedicine technologies to deliver TFs and reprogram cellular phenotypes into immunogenic tumor cell phenotypes. The identification of phenotype drivers or master TFs establishing core regulatory circuits and molecular memory presents potential opportunities for cell reconfiguration [32, 33]. However, TFs are usually nondruggable [1]. We overcame this therapeutic limitation by leveraging emergent RNA technologies and pBAE‐NPs, in combination with immunotherapy, to enhance LCOR expression in tumors and thus promote tumor eradication. These observations lead to a paradigm shift for driver‐cell state or driver‐phenotype therapies in cancer.

mRNA therapies emerge as versatile tools with the potential to transform medicine. Among the different types of mRNA therapy, which are focused mainly on personalized neoantigen vaccines, tumor suppressor replacement therapies of mutated p53 and PTEN have been used in preclinical studies [3, 34]. In our case, we modified LCOR expression, which is not mutated but downregulated in the most aggressive tumor cell populations, including immune‐evasive cancer stem cells [11]. In this regard, it represents an innovative strategy to fine‐tune tumor cell phenotypes based on a TF. This new approach of harnessing mRNA technologies for cancer therapy holds promise in oncology and immuno‐oncology. Moreover, a potential transversal impact on other disciplines of medicine, such as immune‐related disease and regenerative medicine, is also expected.

Polymeric NPs, specifically pBAE polyplexes, show a high transfection efficiency for the *in vivo* expression of diverse encapsulated nucleic acids with low toxicity [35, 36, 37, 38, 39]. In our preclinical models, these pBAE‐NPs effectively transfected a range of breast cancer cells both *in vitro* and *in vivo*, demonstrating their potential as versatile delivery vehicles. pBAE‐NPs offer advantages, including enhanced biocompatibility and biodegradability, chemical versatility, improved endosomal escape, reduced immunogenicity, effective targeting of extrahepatic tissues, suitability for local delivery and ease of manufacture with greater stability [6, 40]. We observed cell‐type dependence of NP uptake across the breast cancer lines tested, which could lead to differential efficiency in distinct types of tumors. Cell‐type‐dependent uptake was previously reported by our group and attributed to the differential endocytic pathways used by specific cell types [25, 26]. In addition, pBAE‐NPs are more prone to disassemble under physiological conditions than other NPs, that is, they efficiently release the mRNA in response to a decrease in pH within the endosomal pathway, thus facilitating endosomal escape. This avoids eventual toxicity caused by NP accumulation, a phenomenon observed in other types of nanomaterial. However, NP disassembly could also compromise the efficacy of systemic administration. Our study shows that local administration reduces systemic toxicity, and the effective dose needed, while also lowering manufacturing costs since specific targeting is not required. Thus, this delivery mode is suitable for local neoadjuvant intratumoral therapies, similar to the use of NPs in protein replacement therapies [41].

A limitation of this study is that the therapeutic strategy was evaluated using local intratumoral administration, which enabled efficient tumor transfection and proof‐of‐concept validation of LCOR transcription‐factor replacement, but does not yet address the challenges associated with systemic delivery or tumor‐specific targeting in disseminated disease. In addition, although our data demonstrate LCOR mRNA translation, APM induction, stemness suppression, immune remodeling, and therapeutic synergy with immune‐checkpoint blockade, the immune characterization was focused on selected pharmacodynamic readouts rather than an exhaustive dissection of all immune populations and mechanisms involved. Therefore, we cannot exclude additional tumor‐cell‐intrinsic and immune microenvironmental effects contributing to the observed therapeutic benefit.

Our results highlight not only the potential of LCOR nanotherapy as a standalone treatment but also when combined with different ICI therapies. Thus, our nanotherapy is designed to efficiently enhance immunogenicity as an optimal complementary therapy to ICI agents designed to restore T cells [11, 12]. In fact, in immuno‐oncology, various combination therapies, including chemotherapy, are used as induction treatments to increase immunogenicity, causing DNA damage and genomic instability [42, 43]. Here we propose *Lcor* mRNA therapy to specifically instigate immunogenic functions that could replace the need to use chemotherapeutic induction treatment strategies. Another breakthrough of our study is that we demonstrate, for the first time, the remarkable performance of *Lcor* mRNA therapy combined with anti‐CTLA4, as well as the preclinical benefit of using *Lcor* mRNA‐loaded RH‐pBAE‐NPs combined with anti‐PD‐L1 blockade. Of note, anti‐CTLA4‐based therapies have received less attention than anti‐PD‐L1 in breast cancer and have not been approved for clinical practice to date. New clinical trials have demonstrated the preliminary efficacy of anti‐CTLA4 blockade in early‐TNBC without the administration of chemotherapy [22]. Therefore, our results indicate the potential benefit of combining our nanotherapy with anti‐CTLA4 blockade, without the need for chemotherapy.

## Conclusions

In conclusion, our study provides proof‐of‐concept that mRNA‐based replacement of the transcription factor LCOR can be used to reprogram tumor cell phenotypes therapeutically. Local delivery of RH‐pBAE‐NPs efficiently introduced Lcor mRNA and restored LCOR activity in breast cancer cells and tumors, promoting antigen presentation, suppressing stemness‐associated features, and modulating the tumor immune microenvironment. Importantly, this local Lcor mRNA nanotherapy enhanced the therapeutic response to PD‐L1 and CTLA4 immune‐checkpoint blockade in the 4T07 preclinical primary tumor model. Together, these findings support transcription factor replacement as a potential strategy to constrain aggressive tumor cell states and increase their susceptibility to immunotherapy, while further development will require evaluation of systemic or tumor‐targeted delivery approaches.

## Conflict of interest

HMRI has filed three patents related to the findings of this study, with T.C.‐T., G.S.‐M., J.A.P., and S.B.‐B. named as coinventors. T.C.‐T. is founder of VIOLET PHARMACEUTICALS, a spin‐off company dedicated to LCOR RNA therapies for oncology.

## Author contributions

TC‐T conceived the study and supervised the project together with CF. TC‐T, CF, and GS‐M designed the study. GS‐M performed most of the *in vitro* experiments and the majority of the *in vivo* experiments, with contributions from JAP, SB‐B, AL‐P, and MS‐F. SB‐B established the tumor immune‐cell isolation and immune‐profiling workflow, performed flow cytometry staining and analysis, and contributed to the additional *in vivo* experiments performed during revision. EH‐M and MS synthesized and biophysically characterized the nanoparticles and performed part of the *in vitro* experiments. CC performed the microscopy imaging. All authors critically reviewed and revised the manuscript for important intellectual content, approved the final version for publication, and agree to be accountable for all aspects of the work.

## Supporting information


**Fig. S1.** Characterization and transfection efficiency of RH‐pBAE nanoparticles. (A) Dynamic light scattering (DLS) analysis of nanoparticles (NPs) was used in this study. Size and polydispersity index (PDI) were measured before and after lyophilization. Data represent mean ± S.E.M. from 3 independent measurements. (B) Flow cytometry analysis of the percentage of several TNBC cells positive for eGFP expression after treatment with mRNA encapsulated in RH‐pBAE‐NPs. Cells were treated with 5 μg of *eGFP* mRNA‐loaded RH‐pBAE‐ NPs for 48 h. Data represent mean % of total gated cells + S.E.M. of *N* = 2 independent experiments for EO771 and EpRAS cell lines and *N* = 3 for AT3, 4T07, EMT6, 66cl4 and 4T1 cell lines. (C) Flow cytometry analysis of the percentage of human TNBC MDA‐MB‐231 and HR + BC MCF7 cells positive for eGFP expression from 5 μg of *eGFP* mRNA‐loaded RH‐pBAE‐NPs. Cells were treated with 5 μg of mRNA‐loaded RH‐pBAE‐NPs for 48 h. Data represents mean % of total gated cells + S.E.M. of *N* = 2 independent experiments. (D) Percentage of *Lcor* mRNA encapsulated by RH‐pBAE‐NPs measured by fluorescence after treatment with the commercial Quant‐it™ RiboGreen kit. Data represents mean + S.E.M of *N* = 3 independent experiments. (E) Flow cytometry analysis of the percentage of TNBC 4T07 and AT3 positive for eGFP expression after treatment with *eGFP* mRNA‐loaded RH‐pBAE‐NPs. Cells were treated with the dose indicated for 12 h. Data represent mean % of total gated cells + S.E.M. from *N* = 3 independent experiments for 4T07 treated with 1 μg·mL^−1^, 4 independent experiments for the rest of 4T07 treatments and AT3 treated with 0.5 μg·mL^−1^, and 5 for the rest of AT3 treatments. Statistical significance: *P < 0.05, **P < 0.01, ***P < 0.001, by Kruskal‐Wallis *H* test, followed by Dunn's *post hoc* test in E.

**Fig. S2.** *Lcor* mRNA design, nanoparticle stability, and functional validation *in vitro*. (A) Schematic representation of the synthetic mRNA used in this study. The Cap1 structure, 5′ and 3′ untranslated regions, polyA tail, and relevant sequences within *Lcor* mRNA are depicted. (B) RT‐qPCR analysis of *Lcor* mRNA levels in TNBC AT3 and 4T07 cells treated with the indicated concentration of mRNA‐loaded RH‐pBAE‐NPs for 12 h. Data are represented as Log_2_(Fc) to the normalized expression of non‐treated control condition. Expression levels were normalized to *Gapdh*. Data represent the mean ± S.E.M. of *N* = 3 independent experiments. (C) Stability of the RH‐pBAE‐NPs over time, measured by FRET efficiency using spectrophotometry. The polymer (R or H) was labeled with Cy5 and the mRNA with Cy3. Stability was assessed at 25 °C in PBS or at 37 °C in 50 mg·mL^−1^ of BSA. Data represent mean + S.E.M. from 3 independent experiments. (D) Schematic representation of the Nanog promoter‐driven dsGFP reporter construct used to assess stem‐like tumor cell populations. Representative flow cytometry contour plots showing Ds‐GFP expression in 4T07 cells 24 h after the indicated treatment conditions. Quantification of mean fluorescence intensity and the percentage of Nanog⁺ tumor cells. Right panels show the frequency of Nanog‐positive (Ds‐GFP⁺) cells within the gated population. Results shown as mean ± S.E.M from 3 independent experiments. (E) RT‐qPCR analysis of co‐stimulatory genes in 4T07 cells treated with 5 μg of the indicated mRNA‐loaded RH‐pBAE‐NPs. Data are represented as Fc to the normalized expression of non‐treated control condition. Expression levels were normalized to *Gapdh*. Data represents the mean ± S.E.M. of *N* = 3 independent experiments. Statistical significance; *P < 0.05, **P < 0.01, ***P < 0.001, Kruskal‐Wallis *H* test, followed by Dunn's *post hoc* test in B, one‐way ANOVA test in C, and unpaired two‐tailed Student's *t*‐test in D and E.

**Fig. S3.** *In vivo* kinetics and immunomodulatory effects of Lcor mRNA nanotherapy. (A) *In vivo* protein expression following treatment with 5 μg of *FLuc* mRNA‐loaded RH‐pBAE‐NPs. 4T07 cells were implanted in the MFP of BALB/c mice. After reaching a size of 40–50 mm^3^, tumors were treated with 5 μg of *FLuc* mRNA‐loaded RH‐pBAE‐NPs. Luminescence was measured at 0, 3, 6, 10, 24 and 48 h post‐administration using IVIS Spectrum. Representative IVIS images and corresponding quantification are shown. Data represent mean ± S.E.M. of *N* = 3–4 mice per group. (B) Tumor volumes at the time of the treatment to evaluate gene expression by RT‐qPCR in orthotopic 4T07 tumors implanted in the mammary fat pad (MFP). Each dot represents one tumor from an individual animal. Data shown as mean ± S.E.M. (C) RT‐qPCR analysis of *Lcor* mRNA levels in tumors formed by AT3 cells implanted in the MFP of C57BL/6 mice. After reaching a size of 40–50 mm^3^, tumors were intratumorally treated with 5 μg of *Lcor* mRNA‐loaded RH‐pBAE‐NPs and harvested 3 h post‐treatment. Data are represented as Log_2_(Fc) to the normalized expression of *FLuc* mRNA treated control condition. Expression levels were normalized to *Gapdh. N* = 3 mice per group (D) RT‐qPCR analysis of APM‐related genes mRNA levels in tumors formed by AT3 cells implanted in the MFP of C57BL/6 mice. After reaching a size of 40–50 mm^3^, tumors were intratumorally treated with 5 μg of *Lcor* mRNA‐loaded RH‐pBAE‐NPs and harvested 3 h post‐treatment. Data are represented as Fc to the normalized expression of *FLuc* mRNA treated control condition. Expression levels were normalized to *Gapdh. N* = 3 mice per group (E) RT‐qPCR analysis of *Lcor* mRNA levels in tumors formed by 4T07 cells implanted in the MFP of BALB/c mice. After reaching a size of 40–50 mm^3^, tumors were intratumorally treated with 5 μg of *FLuc* or *Lcor* mRNA‐loaded RH‐pBAE‐NPs. Data are represented as Log_2_(Fc) to the normalized expression of *FLuc* mRNA treated control condition. Expression levels were normalized to *Gapdh*. *N* = 7 mice per group (F) Flow cytometric quantification of CD8⁺ and CD4⁺ T‐cell infiltration in orthotopic 4T07 tumors after treatment with 5 μg of *FLuc* or *Lcor* mRNA‐loaded RH‐pBAE‐NPs. Frequencies of CD8⁺ and CD4⁺ T cells are shown as percentage of live CD45⁺ tumor‐infiltrating immune cells. Data represent the mean ± S.E.M. Each dot represents one tumor from an individual animal. Statistical significance: *P < 0.05, **P < 0.01, ***P < 0.001, by Mann–Whitney *U* test in B, C and D, and unpaired two‐tailed Student's *t*‐test in E and F.

**Fig. S4.** Body weight and tumor‐growth analysis during anti‐PD‐L1 combination therapy. (A) Relative weight of mice over time in the specified treatments. (B) Tumor volume over time in the specified treatments. Mice were implanted with 4T07 cells in the MFP and tumors treated with 5 μg of the indicated mRNA‐loaded RH‐pBAE‐NPs intratumorally twice a week, and 5 mg·kg^−1^ of anti‐PD‐L1 intraperitoneally once a week. Data represent mean ± S.E.M. from *N* = 5–8 mice per group. Statistical significance: *P < 0.05, **P < 0.01, ***P < 0.001, by linear mixed model analysis with Geisser‐Greenhouse correction, followed by Tukey's multiple comparisons test in A and B. *P*_t4_ = 0.0288; *P*_t5_ = 0.0276.

**Fig. S5.** Tumor‐growth analysis during anti‐CTLA‐4 combination therapy. (A) Tumor volume over time in the specified treatments. Mice were implanted with 4T07 cells in the MFP and tumors treated with 5 μg of mRNA‐loaded RH‐pBAE‐NPs intratumorally twice a week, and 5 mg·kg^−1^ of anti‐CTLA4 intraperitoneally for the first dose, followed by 0.5 mg·kg^−1^ for subsequent doses. Data represent mean ± S.E.M. from *N* = 5–6 mice per group.

**Fig. S6.** Flow‐cytometry gating strategies for tumor and immune‐cell analyses. (A) Gating strategy used to define mCherry+ tumor or mCherry‐ microenvironment cells in Fig. 3A. (B) Gating strategy to define specific immune T‐cell subsets in *in vivo* studies in Fig. 3G.

**Table S1.** Primer sequences used for RT–qPCR analyses.
**Table S2.** Antibodies used for immunofluorescence, immunoblotting, flow cytometry, and *in vivo* experiments.

## Data Availability

In this study, no public datasets or proprietary datasets were used or generated.
