# Supplementary material for: Reprogramming tumor cells with LCOR mRNA nanotherapy to enhance immunotherapy
Source: Mol Oncol. 2026 Oct 15:10.1002/1878-0261.70344. Online ahead of print. doi: 10.1002/1878-0261.70344 (PMC13670883; doi:10.1002/1878-0261.70344)
Supplement: Supplementary file 1 [file MOL2-9999-0-s006.docx]

**Supplementary FIGURE LEGENDS**

**Figure S1. Characterization and transfection efficiency of RH-pBAE nanoparticles.**

(a) Dynamic light scattering (DLS) analysis of nanoparticles (NPs) was used in this study. Size and polydispersity index (PDI) were measured before and after lyophilization. Data represent mean ± S.E.M. from 3 independent measurements.
(b) Flow cytometry analysis of the percentage of several TNBC cells positive for eGFP expression after treatment with mRNA encapsulated in RH-pBAE-NPs. Cells were treated with 5 µg of *eGFP* mRNA-loaded RH-pBAE- NPs for 48 h. Data represent mean % of total gated cells + S.E.M. of N = 2 independent experiments for EO771 and EpRAS cell lines and N = 3 for AT3, 4T07, EMT6, 66cl4 and 4T1 cell lines.
(c) Flow cytometry analysis of the percentage of human TNBC MDA-MB-231 and HR+BC MCF7 cells positive for eGFP expression from 5 µg of *eGFP* mRNA-loaded RH-pBAE-NPs. Cells were treated with 5 µg of mRNA-loaded RH-pBAE-NPs for 48 h. Data represents mean % of total gated cells + S.E.M. of N = 2 independent experiments.
(d) Percentage of *Lcor* mRNA encapsulated by RH-pBAE-NPs measured by fluorescence after treatment with the commercial Quant-it^TM^ RiboGreen kit. Data represents mean + S.E.M of N = 3 independent experiments.
(e) Flow cytometry analysis of the percentage of TNBC 4T07 and AT3 positive for eGFP expression after treatment with *eGFP* mRNA-loaded RH-pBAE-NPs. Cells were treated with the dose indicated for 12 h. Data represent mean % of total gated cells + S.E.M. N = 3 independent experiments for 4T07 treated with 1 µg/mL, 4 independent experiments for the rest of 4T07 treatments and AT3 treated with 0.5 µg/mL, and 5 for the rest of AT3 treatments. Statistical significance: *p<0.05, **p<0.01, ***p<0.001, by Kruskal-Wallis H test, followed by Dunn’s post hoc test in E.

**Figure S2. Lcor mRNA design, nanoparticle stability, and functional validation in vitro.**

(a) Schematic representation of the synthetic mRNA used in this study. The Cap1 structure, 5’ and 3’ untranslated regions, polyA tail, and relevant sequences within *Lcor* mRNA are depicted.
(b) RT-qPCR analysis of *Lcor* mRNA levels in TNBC AT3 and 4T07 cells treated with the indicated concentration of mRNA-loaded RH-pBAE-NPs for 12 h. Data are represented as Log_2_(Fc) to the normalized expression of non-treated control condition. Expression levels were normalized to *Gapdh*. Data represent the mean ± S.E.M. of N = 3 independent experiments.
(c) Stability of the RH-pBAE-NPs over time, measured by FRET efficiency using spectrophotometry. The polymer (R or H) was labeled with Cy5 and the mRNA with Cy3. Stability was assessed at 25ºC in PBS or at 37ºC in 50 mg/mL of BSA. Data represents mean + S.E.M. from 3 independent experiments.
(d) Schematic representation of the Nanog promoter-driven dsGFP reporter construct used to assess stem-like tumor cell populations. Representative flow cytometry contour plots showing dsGFP expression in 4T07 cells 24h after the indicated treatment conditions. Quantification of mean fluorescence intensity and the percentage of Nanog⁺ tumor cells. Right panels show the frequency of Nanog-positive (dsGFP⁺) cells within the gated population. Results shown as mean ± S.E.M from 3 independent experiments.
(e) RT-qPCR analysis of co-stimulatory genes in 4T07 cells treated with 5 µg of the indicated mRNA-loaded RH-pBAE-NPs. Data are represented as Fc to the normalized expression of non-treated control condition. Expression levels were normalized to *Gapdh*. Data represents the mean ± S.E.M. of N = 3 independent experiments. Statistical significance; *p<0.05, **p<0.01, ***p<0.001, Kruskal-Wallis H test, followed by Dunn’s post hoc test in B, one-way ANOVA test in C, and unpaired two-tailed Student’s t-test in D and E.

**Figure S3. In vivo kinetics and immunomodulatory effects of Lcor mRNA nanotherapy.**

(a) *In vivo* protein expression following treatment with 5 µg of *FLuc* mRNA-loaded RH-pBAE-NPs. 4T07 cells were implanted in the MFP of Balb/C mice. After reaching a size of 40-50 mm^3^, tumors were treated with 5 µg of *FLuc* mRNA-loaded RH-pBAE-NPs. Luminescence was measured at 0, 3, 6, 10, 24 and 48 h post-administration using IVIS Spectrum. Representative IVIS images and corresponding quantification are shown. Data represents mean ± S.E.M. N = 3–4 mice per group.
(b) Tumor volumes at the time of the treatment to evaluate gene expression by RT-qPCR in orthotopic 4T07 tumors implanted in the mammary fat pad (MFP). Each dot represents one tumor from an individual animal. Data shown as mean ± S.E.M.
(c) RT-qPCR analysis of *Lcor* mRNA levels in tumors formed by AT3 cells implanted in the MFP of C57BL/6 mice. After reaching a size of 40-50 mm^3^, tumors were intratumorally treated with 5 µg of *Lcor* mRNA-loaded RH-pBAE-NPs and harvested 3 h post-treatment. Data are represented as Log_2_(Fc) to the normalized expression of *FLuc* mRNA treated control condition. Expression levels were normalized to *Gapdh.* N = 3 mice per group
(d) RT-qPCR analysis of APM-related genes mRNA levels in tumors formed by AT3 cells implanted in the MFP of C57BL/6 mice. After reaching a size of 40-50 mm^3^, tumors were intratumorally treated with 5 µg of *Lcor* mRNA-loaded RH-pBAE-NPs and harvested 3 h post-treatment. Data are represented as Fc to the normalized expression of *FLuc* mRNA treated control condition. Expression levels were normalized to *Gapdh.* N = 3 mice per group
(e) RT-qPCR analysis of *Lcor* mRNA levels in tumors formed by 4T07 cells implanted in the MFP of Balb/c mice. After reaching a size of 40-50 mm^3^, tumors were intratumorally treated with 5 µg of *FLuc* or *Lcor* mRNA-loaded RH-pBAE-NPs. Data are represented as Log_2_(Fc) to the normalized expression of *FLuc* mRNA treated control condition. Expression levels were normalized to *Gapdh.* N = 7 mice per group
(f) Flow cytometric quantification of CD8⁺ and CD4⁺ T-cell infiltration in orthotopic 4T07 tumors after treatment with 5 µg *FLuc* or *Lcor* mRNA-loaded RH-pBAE-NPs. Frequencies of CD8⁺ and CD4⁺ T cells are shown as percentage of live CD45⁺ tumor-infiltrating immune cells. Each dot represents one tumor from an individual mouse; data represents the mean ± S.E.M. Each dot represents one tumor from an individual animal. Statistical significance; *p<0.05, **p<0.01, ***p<0.001, by Mann-Whitney U test in B, C and D, and unpaired two-tailed Student’s t-test in E and F.

**Figure S4. Body weight and tumor-growth analysis during anti-PD-L1 combination therapy.**

(a) Relative weight of mice over time in the specified treatments.
(b) Tumor volume over time in the specified treatments. Mice were implanted with 4T07 cells in the MFP and tumors treated with 5 µg of the indicated mRNA-loaded RH-pBAE-NPs intratumorally twice a week, and 5 mg/kg of anti-PD-L1 intraperitoneally once a week. Data represent mean ± S.E.M. from N = 5–8 mice per group. Statistical significance: *p<0.05, **p<0.01, ***p<0.001, by linear mixed model analysis with Geisser-Greenhouse correction, followed by Tukey’s multiple comparisons test in A and B. *p_t4_*=0.0288; *p_t5_*=0.0276.

**Figure S5. Tumor-growth analysis during anti-CTLA-4 combination therapy.**

(a) Tumor volume over time in the specified treatments. Mice were implanted with 4T07 cells in the MFP and tumors treated with 5 µg of mRNA-loaded RH-pBAE-NPs intratumorally twice a week, and 5 mg/kg of anti-CTLA4 intraperitoneally for the first dose, followed by 0.5 mg/kg for subsequent doses. Data represent mean ± S.E.M. from N = 5–6 mice per group.

**Figure S6. Flow-cytometry gating strategies for tumor and immune-cell analyses.**

(a) Gating strategy used to define mCherry+ tumor or mCherry- microenvironment cells in Figure 3A.
(b) Gating strategy to define specific immune T cell subsets in *in vivo* studies in Figure 3G.

**Supplementary Table 1. Primer sequences used for RT–qPCR analyses**

| Primer Name | Sequence |
| --- | --- |
| mGapdh fw | AGGTCGGTGTGAACGGATTTG |
| mGapdh rev | TGTAGACCATGTAGTTGAGGTCA |
| mLcor fw | GCTCATGGCTGACCAAGACT |
| mLcor rev | GAATGGCTCAGGGAAGTGCT |
| mB2m fw | CTCGGTGACCCTGGTCTTTC |
| mB2m rev | TTGAGGGGTTTTCTGGATAGCA |
| mH-2K fw | TCCACTGTCTCCAACATGGC |
| mH-2K rev | TCCCCTCCTTTTCCACCTGT |
| mPsmb8 fw | GCCACTGCAGGGAGTTACAT |
| mPsmb8 rev | ATACAACCTGCACTCCTTGGC |
| mPsmb9 fw | GCTCTGGAAGTAAACCAAGAGAC |
| mPsmb9 rev | TGGTTGTCTTCTCACCAGCTT |
| mTap1 fw | AAGTGCATCAGTCACTGGCA |
| mTap1 rev | ATCGCCGATAAGGCCTCAAG |
| mTap2 fw | ACTGTGAGGACGCTCAAGTG |
| mTap2 rev | CCCTTTTTCCCCGATTTCTGTG |
| mSox2 fw | AGAGCTAGACTCCGGGCGA |
| mSox2 rev | ACCCAGCAAGAACCCTTTC |
| mOct4 fw | CACCATCTGTCGCTTCGAGG |
| mOct4 rev | AGGGTCTCCGATTTGCATATCT |
| mNanog fw | TCTTCCTGGTCCCCACAGTTT |
| mNanog rev | GCAAGAAATAGTTCTCGGGATGAA |
| mZeb1 fw | GCTGGCAAGACAACGTGAAAG |
| mZeb1 rev | GCCTCAGGATAAATGACGG |
| mPdl1 fw | GCTCCAAAGGACTTGTACGTG |
| mPdl1 rev | TGATCTGAAGGGCAGCATTTC |
| mCd80 fw | ACCCCCAACATAACTGAGTCT |
| mCd80 rev | TTCCAACCAAGAGAAGCGAGG |
| mCd86 fw | TGTTTCCGTGGAGACGCAAG |
| mCd86 rev | TTGAGCCTTTGTAAATGGGCA |

**Supplementary Table 2. Antibodies used for immunofluorescence, immunoblotting, flow cytometry, and in vivo experiments**

| Antibody name | Source | ID |
| --- | --- | --- |
| IF and WB: Rb pAB to HA tag | Abcam | AB9110 |
| IF: Goat anti-Rabbit IgG (H+L) cross-Adsorbed secondary antibody AF 555 | Invitrogen | A21428 |
| IF: Ms mAb CD8 alpha | Abcam | AB17147 |
| IF: mAb mouse CD4 | Thermo Fisher | 4SM95 |
| FC: APC anti-mouse H-2K^d^ | BioLegend | 116620 |
| FC: APC Mouse IgG2a, κ Isotype Ctrl | BioLegend | 400220 |
| FC: OVA257-264 (SIINFEKL) peptide bound to H-2Kb, APC | eBioscience | 17-5743-82 |
| FC: Mouse IgG1 kappa Isotype Control (P3.6.2.8.1), APC | eBioscience | 17-4714-82 |
| FC: cFluor v547 anti CD45 | Cytek | R7-20571 |
| FC: BUV395 CD8 | BD Bioscience | 565968 |
| FC: BV750 CD4 | Biolegend | 100467 |
| FC: AF488 GZMB | Biolegend | 396423 |
| FC: PE-CF594 CD69 | BD Bioscience | 562455 |
| FC: APC PD1 | Biolegend | 135210 |
| WB: Goat Anti-Rabbit IgG H&L (HRP) | Abcam | AB6721 |
| In vivo: *inVivo*MAB anti-mouse PD-L1 (B7-H1) | Bioxcell | BE0101 |
| In vivo: *InVivo*MAb anti-mouse CTLA4 (CD152) | Bioxcell | BE0131 |
| In vivo: *InVivo*MAb rat IgG2b isotype control, anti-keyhole limpet hemocyanin | Bioxcell | BE0900 |
| In vivo: *InVivo*MAb polyclonal Syrian hamster IgG | Bioxcell | BE0087 |
